# Development, Evaluation, and Implementation of a Cost-Effective, Novel Lateral Canthotomy Simulation Model for Training Emergency Medicine Physicians

**DOI:** 10.7759/cureus.73833

**Published:** 2024-11-17

**Authors:** Maryam Al Ali, Omar Noori, Ayesha Maklai

**Affiliations:** 1 Emergency Department, Rashid Hospital, Dubai, ARE; 2 Emergency Department, Fakeeh University Hospital, Dubai, ARE

**Keywords:** cost-effective medical training, emergency medicine training, lateral canthotomy, orbital compartment syndrome, simulation model

## Abstract

This study proposes a new, low-cost lateral canthotomy simulation model for the purpose of improving training among emergency medicine physicians. This model is important for the rehearsal and honing of skills necessary in such a critical procedure, in cases where this procedure needs to be done in a sensitive situation where an ophthalmologist is not readily available. We surveyed 15 emergency residents before and after the training on comfort and proficiency in performing lateral canthotomy with a standardized Likert scale. Results show a significant increase in comfort and familiarity with the procedure post training, with average comfort levels increasing from 2.4 at pre-training to approximately 3.87 at post training. These findings would, therefore, indicate that the novel simulation model effectively bridges the proficiency gap and should then translate into better preparedness with the potential to improve patient outcomes in emergency settings.

## Introduction

Orbital compartment syndrome (OCS) is a sight-threatening emergency characterized by increased intraorbital pressure, which can compromise optic nerve perfusion and lead to permanent vision loss if not promptly managed [1]. Orbital compartment syndrome often arises from trauma, hemorrhage, infection, or inflammation causing rapid accumulation of fluid or blood within the confined orbital space. Clinical identification of OCS involves recognizing signs such as proptosis (forward displacement of the eye), decreased visual acuity, afferent pupillary defect, ophthalmoplegia (restricted eye movements), elevated intraocular pressure, and periorbital swelling or ecchymosis [2]. Due to the relative rarity of OCS, timely recognition and the ability to perform a lateral canthotomy are crucial to prevent permanent vision loss [3].

Lateral canthotomy is a vision-saving emergency procedure often required to relieve rising intraocular pressure in cases of OCS [4]. The procedure involves several critical steps that are as follows: 1. Aseptic preparation: clean and prepare the periocular area using antiseptic solutions to prevent infection; 2. Identification of anatomical landmarks: locate the lateral canthus, the outer corner where the upper and lower eyelids meet; 3. Hemostat application: apply a hemostat horizontally to the lateral canthus to crush the lateral canthal tendon for 15-30 seconds. This step aids in hemostasis and marks the area for incision; 4. Incision (canthotomy): make a horizontal incision approximately 1-2 cm in length at the lateral canthus, cutting through the skin and orbicularis oculi muscle to access the lateral canthal tendon; 5. Lateral cantholysis: using scissors, cut the inferior crus of the lateral canthal tendon to release the lower eyelid, thereby relieving orbital pressure. If necessary, the superior crus may also be released; 6. Reassessment: evaluate the intraocular pressure and ocular function to determine the effectiveness of decompression.

Throughout the procedure, care must be taken to avoid injury to surrounding structures such as the globe and lacrimal gland. Immediate reassessment is essential to ensure that the decompression has alleviated the signs of OCS [5].

However, real-world opportunities for hands-on practice can be scarce due to the rarity of the condition, resulting in a proficiency gap among emergency medicine practitioners [6]. Although various simulation models exist, each comes with its own set of advantages and disadvantages [7]. The goal of this study is to develop an affordable, easily assembled simulation model to bridge this proficiency gap and expand training opportunities for emergency physicians.

## Technical report

Methods

**Figure 1:**
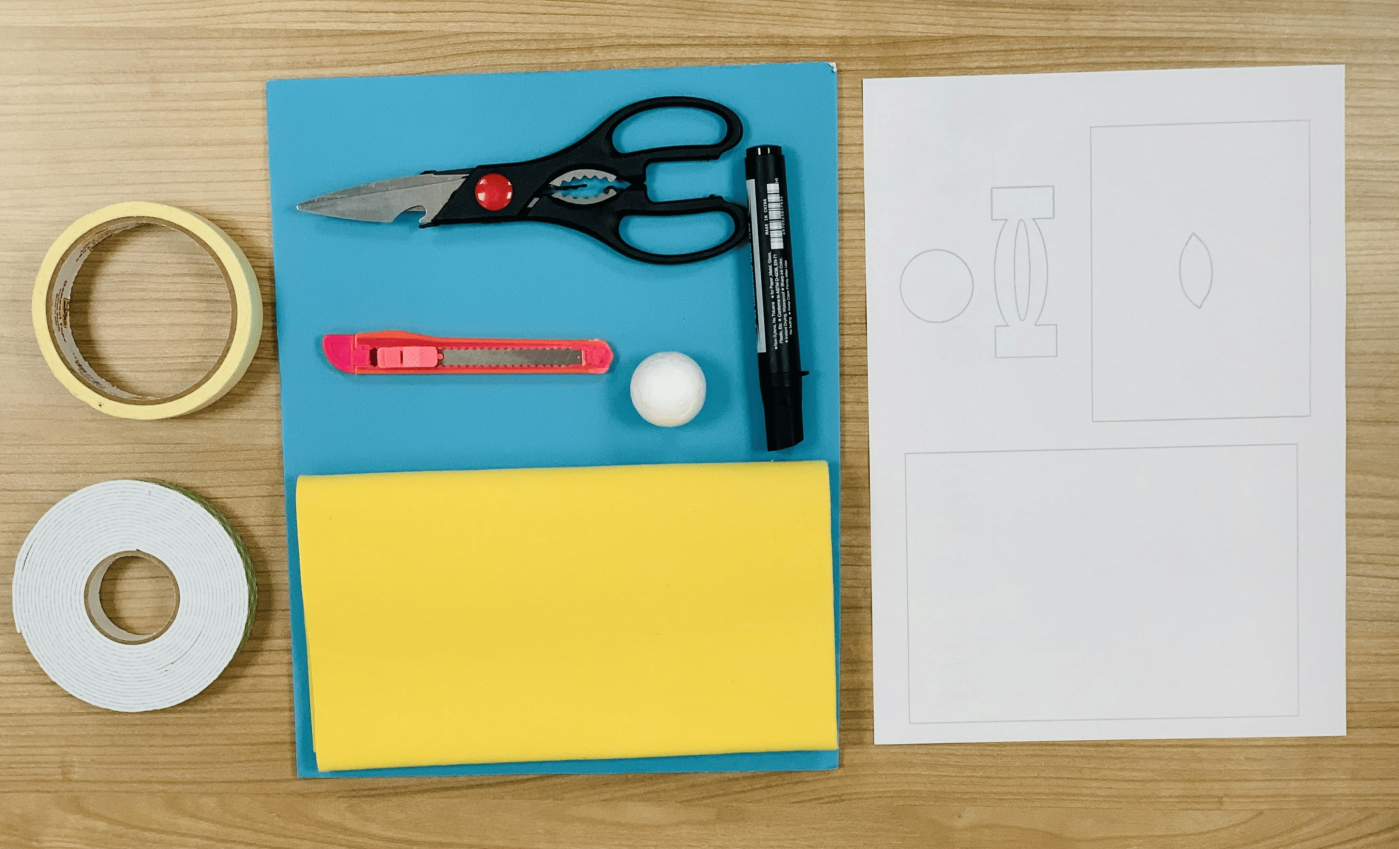
Materials used for constructing the lateral canthotomy simulation model The components include the orbit (soft foam ball, 2.5 cm), the lateral canthal ligament (adhesive tape), the skin (microfiber cloth), and additional tools such as a marking pen, double-sided adhesive tape, scissors, and a scalpel.

All materials should be prepared before starting to create the model of a lateral canthotomy. Materials used in the building of this model are presented in Figure 1.

**Figure 2:**
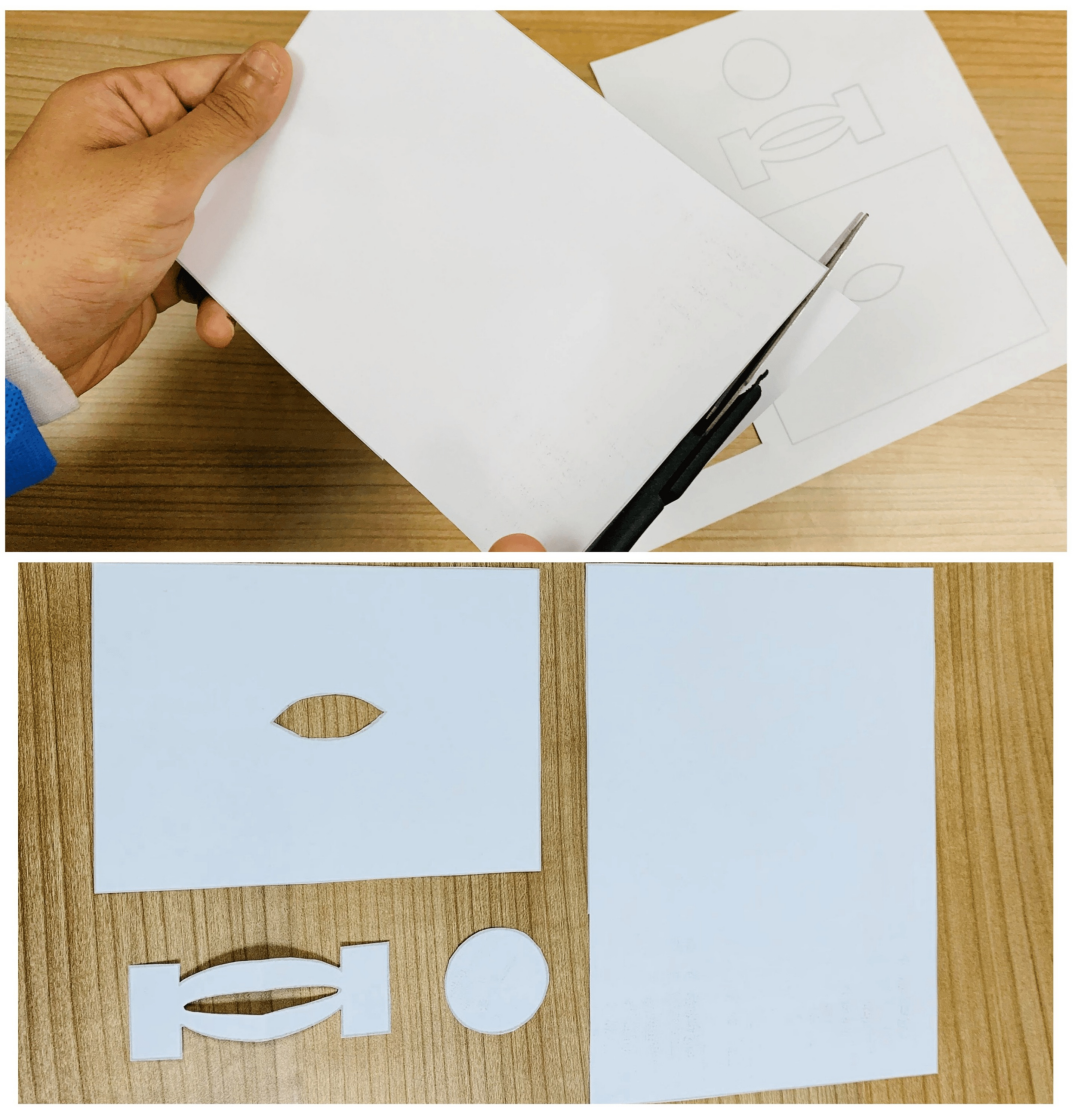
Cutting the model components After printing out the assembly model, each part of the figure is cut separately following the outline. This figure shows the process of cutting the templates and the final pieces that will be used in constructing the lateral canthotomy simulation model.

Gathering is followed by the preparation of components of the model. After printing out the assembly template (Appendix B), every part needs to be cut out along the lines very carefully for the correct assembly, as shown in Figure 2.

**Figure 3:**
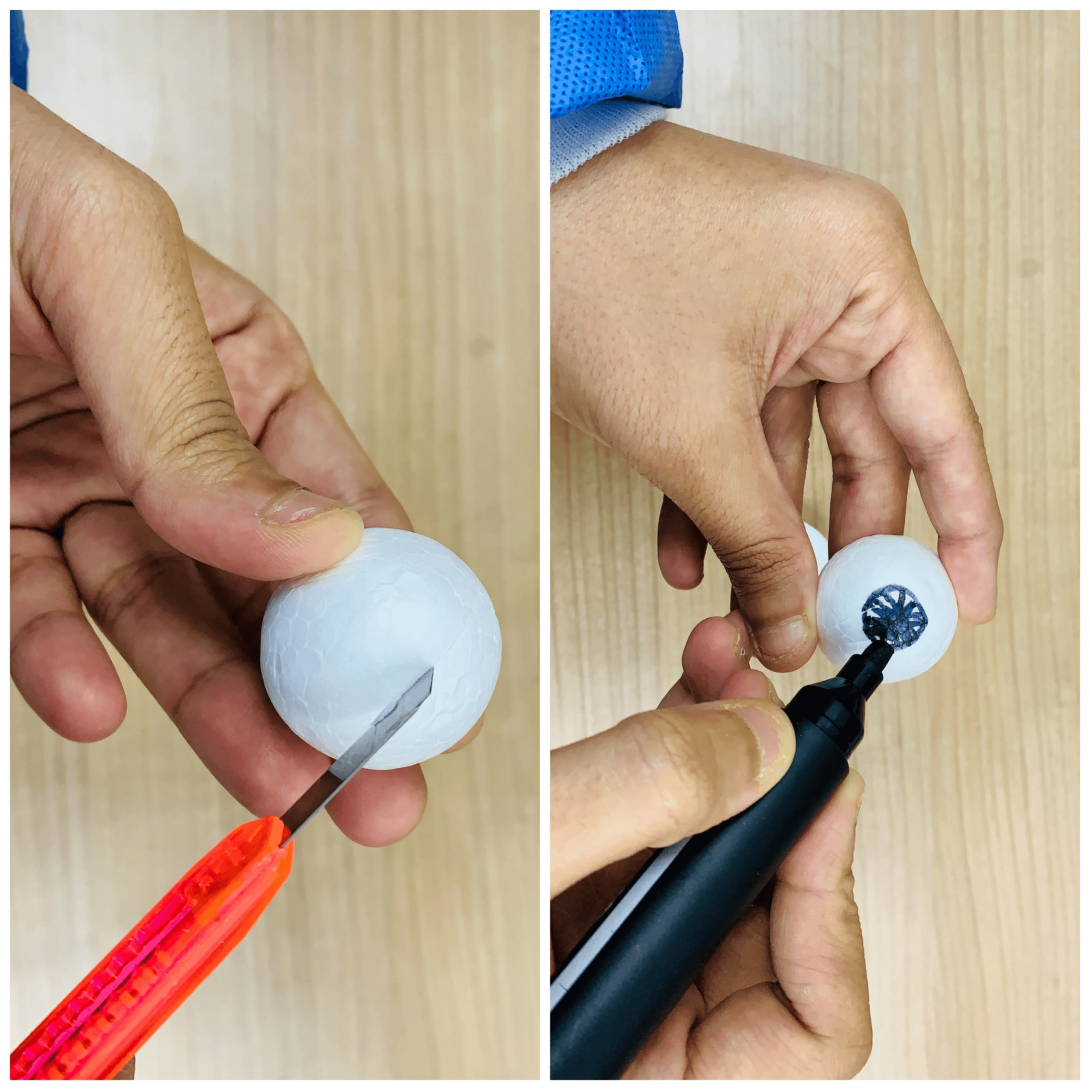
Preparing the orbit (part A) A straight line was drawn through the midline of the soft foam ball to divide it into two halves. An iris is then marked on the curved surface using a marker, making sure the details are appropriate for the simulation. Adequate time is allowed for the marker ink to dry.

The next step involved making the orbit (part A), represented in Figure 3, which illustrates how to create the orbit using the soft foam ball.

**Figure 4:**
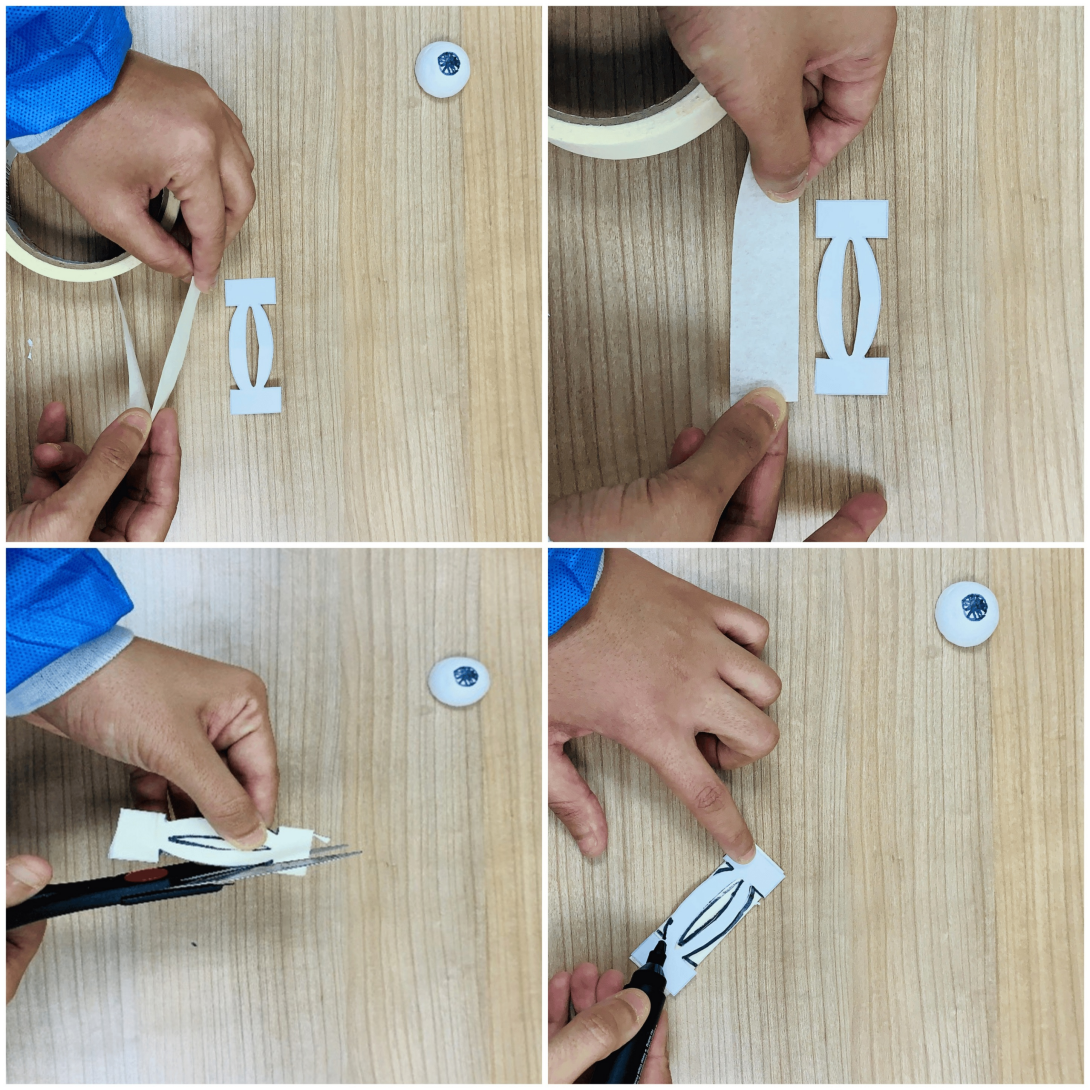
Constructing the lateral canthal tendon (part B) Using part B from the assembly template, the adhesive tape is measured and cut to the appropriate length. Five layers of tape are stacked up one on top of the other. With the stencil as a guide, the shape using a marker is outlined, and the excess tape along the edges is carefully trimmed to match the template.

The following step is the creation of the lateral canthal tendon (part B), using tape adhesives to form a flexible but durable model part while ensuring an accurate representation of the tendon as shown in Figure 4.

**Figure 5:**
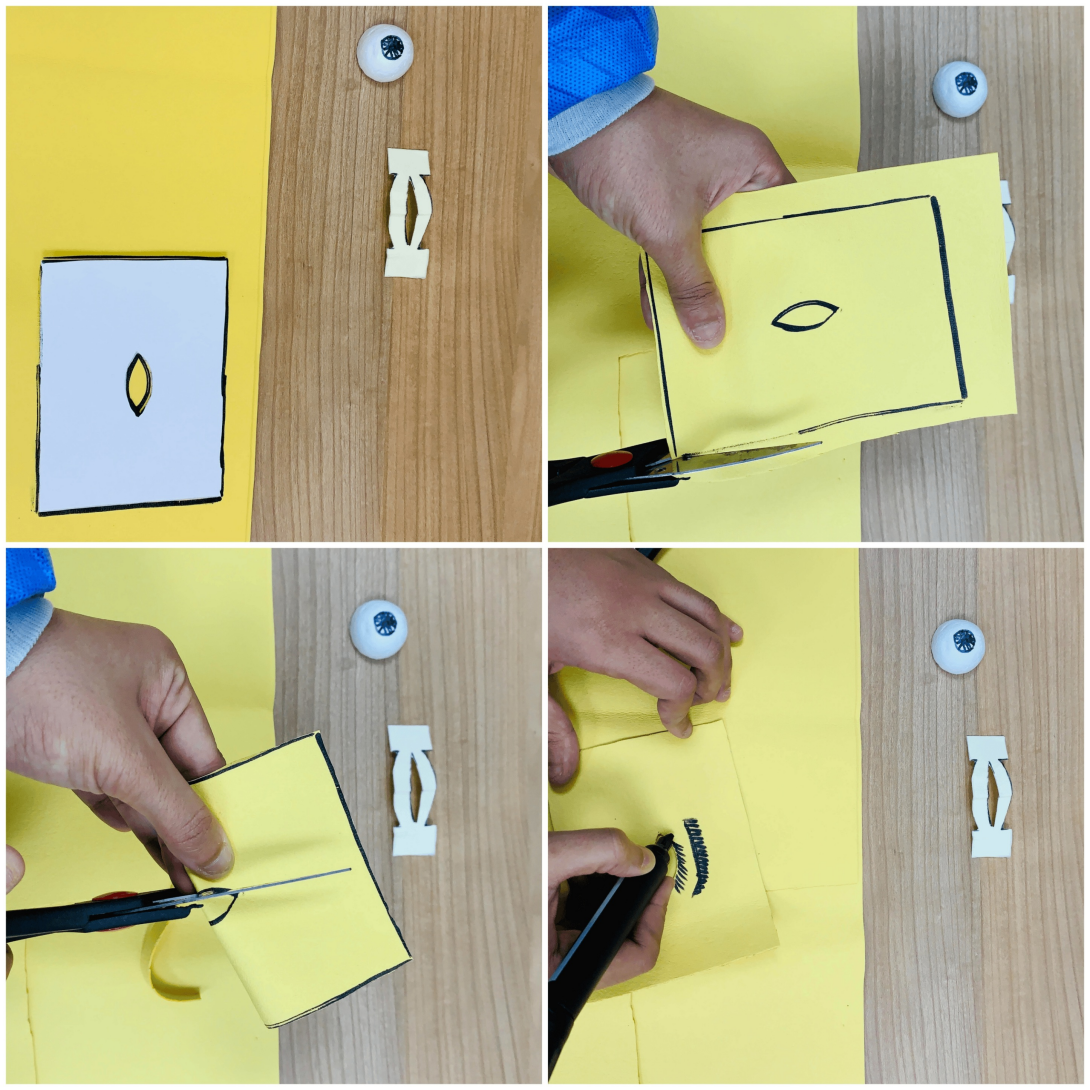
Preparing the skin and eyelids (part C) Using part C from the assembly template, the dimensions of the microfiber cloth are measured and cut to size. An oval opening in the center is created to represent the eyelid. An eyebrow is drawn above the opening to help the learner differentiate the lateral from the medial aspect of the model.

Further preparation of the skin and eyelids (part C) provides a more realistic surface for practicing the procedure, as shown in Figure 5. This part adds a degree of realism in terms of anatomical detail to the simulation.

**Figure 6:**
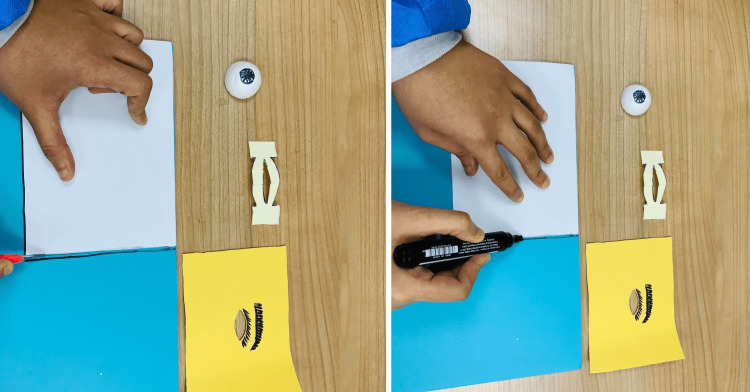
Preparing the base of the model (part D) Using part D from the assembly template, the outline onto the hard foam is traced. The foam is carefully cut along the traced lines to create the base that would support the model components.

The next step in constructing the model is to prepare the base (part D), which provides the foundational structure for the entire simulation model, which is represented in Figure 6.

**Figure 7:**
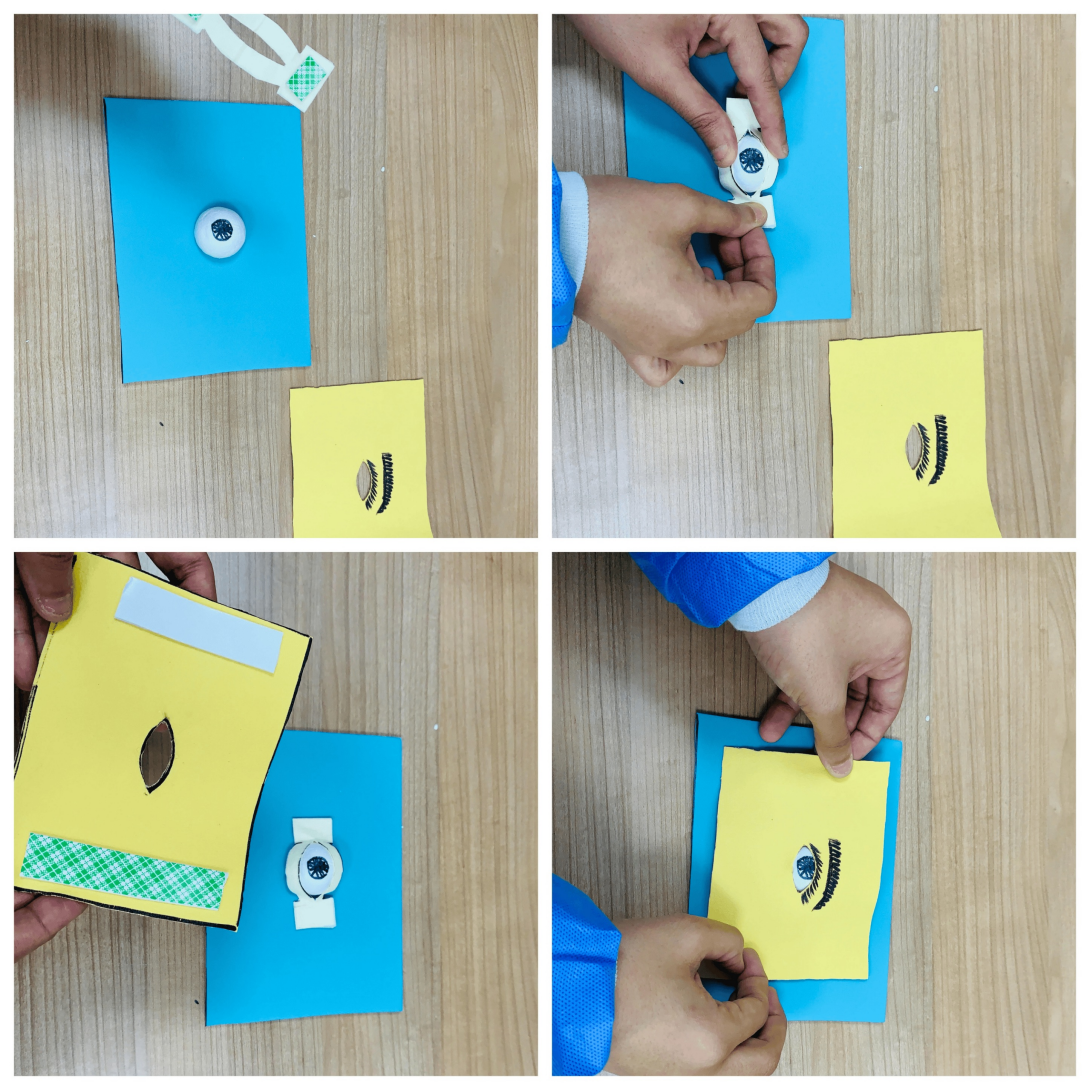
Assembling the lateral canthotomy simulation model The soft foam ball (the orbit) is attached to the center of the base using double-sided adhesive tape. The lateral canthal tendon (part B) is fixed on both sides of the foam ball using the same tape. If needed, cutting along the dotted lines is done to widen the space between the superior and inferior tendons. The microfiber layer (part C) representing the skin and eyelid on top is placed securing it with adhesive tape along the edges.

With all parts of the model prepared, the final step is to assemble them together to complete the lateral canthotomy simulation model, as in Figure 7.

**Figure 8:**
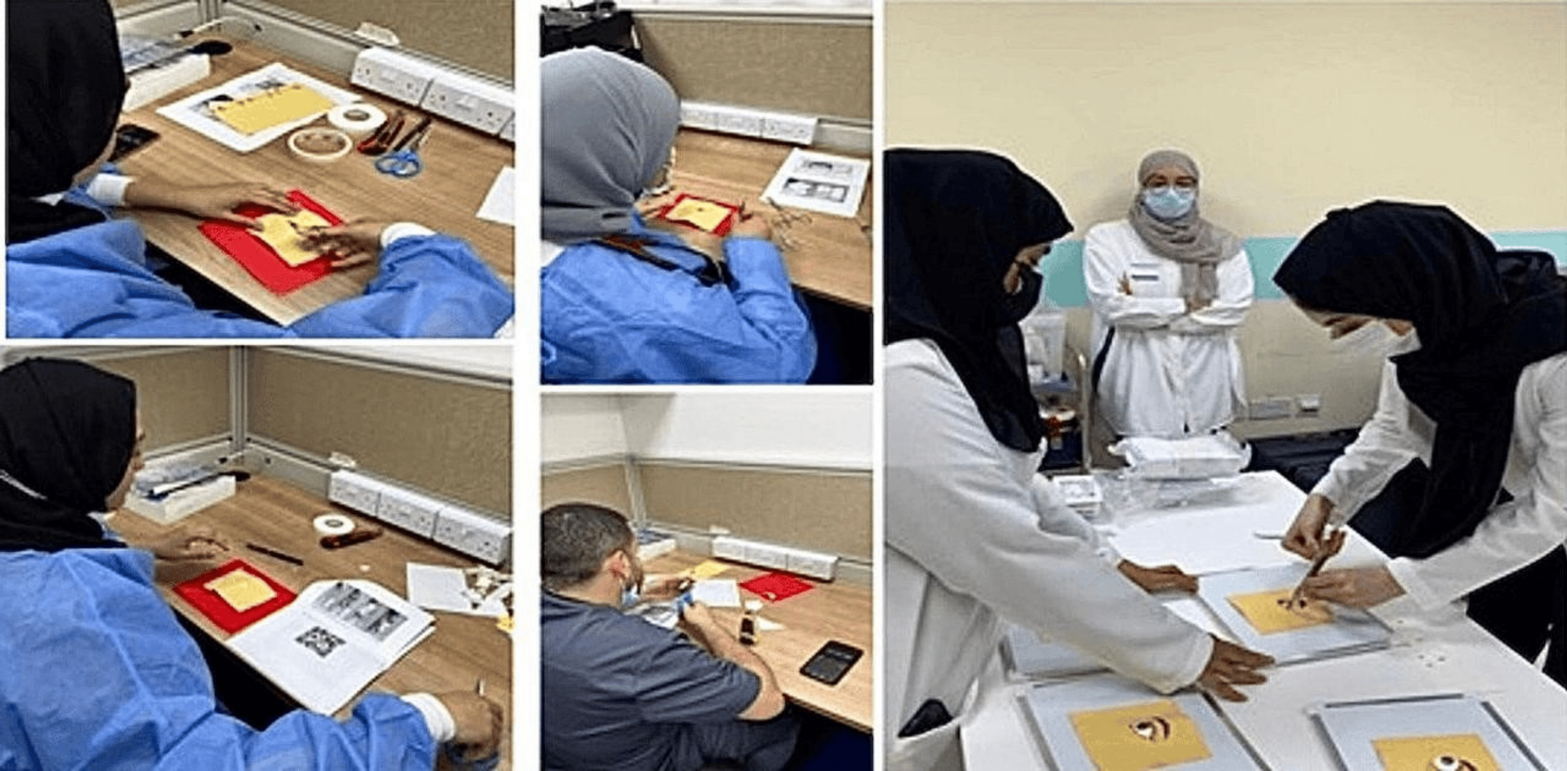
Residents making and using their models

As seen in Figure 8, residents were actively involved in creating and utilizing their own models during the training session.

In addition to the figures, a comprehensive video explanation of the process is available and can be seen in Videos 1-2.

**Video 1:** Model assembly

**Video 2:** Procedure demonstration

To assess the effectiveness of the model, we conducted a pre- and post-training survey gauging the comfort level of emergency physicians in performing the lateral canthotomy procedure (Appendix A).

Results

The participants' age range was from the late twenties onwards, with an average of 28.1 years and a standard deviation of 2.4 years, indicating a small variation in age. With regard to the distribution of gender, 86.7% of the respondents (n = 13) identified as females, and 13.3% identified themselves as males.

The population consisted of residents from classes of the first to the fifth year of residency. First- and fifth-year residents each represented the largest proportion of participants, at 26.7% (n = 4), while fourth-year residents constituted 20% (n = 3). The graduate and third-year residents made up 13.3% each of the group (n = 2).

Prior to using the simulation model, participants exhibited varying levels of experience with the lateral canthotomy procedure. Notably, 66.7% (n = 10) had no prior experience, whereas 33.3% (n = 5) had practiced the procedure on a different simulation model.

Comfort Levels Before and After the Simulation

Participants rated their comfort level with the procedure before and after the simulation on a scale from one (very uncomfortable) to five (very comfortable). Table 1 presents the distribution of comfort levels pre- and post simulation. Before the simulation, the majority of participants rated their comfort level as neutral (three) or lower, with a mean comfort level of 2.4 (SD = 0.91). After practicing with the model, there was a significant shift towards higher comfort levels, with a mean comfort level of 3.87 (SD = 0.74). This indicates an overall improvement in confidence post simulation.

**Table 1:** Participants’ comfort levels before and after simulation training (n=15) SD: standard deviation

Comfort level rating	Pre-simulation n (%)	Post simulation n (%)
1 (very uncomfortable)	4 (26.7%)	0 (0%)
2	3 (20.0%)	0 (0%)
3 (neutral)	7 (46.7%)	5 (33.3%)
4	0 (0%)	7 (46.7%)
5 (very comfortable)	1 (6.7%)	3 (20.0%)
Mean (SD)	2.4 (0.91)	3.87 (0.74)
Total	15 (100%)	15 (100%)

Ease of Assembly and Reusability Ratings

Participants rated the ease of assembly and the model's reusability on a scale from one (very easy) to five (very difficult). Table 2 shows the distribution of these ratings. The mean rating for ease of assembly was 2.13 (SD = 0.83), indicating that overall, participants found the assembly process easy. For reusability, the mean rating was 2.73 (SD = 1.62), suggesting moderate ease of reuse, though responses varied more widely compared to assembly ease.

**Table 2:** Participants’ ratings on ease of assembly and reusability of the simulation model (n=15)

Rating scale	Ease of assembly n (%)	Reusability n (%)
1 (very easy)	4 (26.7%)	4 (26.7%)
2 (easy)	5 (33.3%)	5 (33.3%)
3 (neutral)	0 (0%)	1 (6.7%)
4 (difficult)	6 (40.0%)	2 (13.3%)
5 (very difficult)	0 (0%)	3 (20.0%)
Mean (SD)	2.13 (0.83)	2.73 (1.62)
Total	15 (100%)	15 (100%)

Time to Assemble the Simulation Model

The time participants took to assemble the simulation model varied, with an average of 18.9 minutes (range five to 40 minutes). The most frequently reported assembly times were 17 minutes, 21 minutes, and 22 minutes, each reported by 13.3% (n = 2) of participants.

The results demonstrate that the simulation model effectively enhanced participants' comfort levels with the lateral canthotomy procedure. The mean comfort level increased from 2.4 pre-simulation to 3.87 post-simulation, highlighting a significant improvement in confidence. Before the simulation, 46.7% (n = 7) of participants rated their comfort level as neutral, and 46.7% (n = 7) felt uncomfortable (ratings of one or two). Post simulation, 66.7% (n = 10) rated their comfort level as comfortable or very comfortable (ratings of four or five).

Regarding the model's assembly, the mean ease-of-assembly rating was 2.13, suggesting that most participants found it easy to assemble. However, 40% (n = 6) rated assembly difficulty as four, indicating room for improvement in simplifying the assembly process. The reusability mean rating was 2.73, with 60% (n = 9) finding it easy or very easy to reuse the model. Nevertheless, 33.3% (n = 5) found reusability to be difficult or very difficult (ratings of four or five), suggesting that enhancing the model's durability could benefit future training sessions.

Overall, the implementation of the cost-effective lateral canthotomy simulation model appears to be a valuable tool for training emergency medicine physicians, improving their confidence and potentially leading to better patient care outcomes in real-life scenarios [8].

Financial aspects

When testing the feasibility of a surgical training model, financial viability comes to the center stage. The cost-effectiveness of our lateral canthotomy model was paramount in its advantage, given the efficiency, reusability, and ease of low-cost material to build such a model.

The total cost to build the model is around 50 United Arab Emirates dirhams (AED) or 13 US dollars (USD). These include hard foam, which is going to cost 15 AED or 4 USD; a soft foam ball at 3 AED or 1 USD; adhesive tape, 2 AED or 0.5 USD; microfiber cloth, 15 AED or 4 USD; glue, 10 AED or 3 USD; and double-sided adhesive tape, 10 AED or 3 USD. This low price per unit means the model will be economical for hospitals and medical centers that have a low budget for training purposes.

In addition to the low material costs, the model is quick to assemble, with an average assembly time of 30 minutes, reducing labor expenses and allowing more time to focus on training itself. The simplicity of the assembly process also eliminates the need for highly specialized personnel, further contributing to cost savings [9].

A major financial benefit of our model is its reusability. After initial use, only the skin layers and lateral ligaments need replacement, a process that takes less than two minutes. This ability to reuse the model multiple times in a single training session significantly reduces overall costs, enhancing its cost-effectiveness.

Besides that, this model has huge long-term financial impacts. By offering physicians a cost-efficient manner of practicing a critical but infrequently performed procedure, our model can reduce surgical complications, decrease patient morbidity, and overall decrease the burden on healthcare systems financially [10].

In summary, the financial benefits of our model are considerable. Its low cost, ease of assembly, and reusability make it a cost-effective training solution. A thorough cost analysis underscores the model’s financial viability, making it an attractive option for institutions aiming to optimize both training efficiency and budget.

## Discussion

This is a presentation of a novel lateral canthotomy simulation model that has great potential, as has already been determined, for improving resident training and preparation. The fact that the model is easy to assemble and that participants' confidence was increased manifold after the training is indicative of the effectiveness of the model and what actual benefits it will provide in medical education.

While the model has its benefits, it also suffers from a number of limitations common in all simulation-based training models. Compared to high-fidelity models like 3D-printed devices, our low-cost model offers a practical alternative in resource-limited settings, combining affordability with effective training outcomes [11]. For one, it does not mimic real-life physiological scenarios such as bleeding, which reduces realism and the overall effectiveness of such training. This is one particular limitation that future research should concentrate on to enhance this model's fidelity and usability even further.

When compared to existing simulation models, such as high-fidelity mannequins like SimMan by Laerdal and specialized task trainers like the Orbital Trauma Simulator, our lateral canthotomy model emerges as a practical and cost-effective alternative, particularly advantageous in resource-limited settings. It is easy and cheap; thus, it is accessible to a wide range of medical institutions, which allows its wide application. The model, because it would afford emergency physicians the opportunity for hands-on practice, is likely to boost their confidence and skills, hence improving patient outcomes in time-sensitive critical situations [4]. Additionally, the simulation incorporates potential complications such as ocular hemorrhage, inadvertent injury to the globe, and damage to surrounding tissues. To address ocular hemorrhage, the model allows physicians to practice precise incision techniques and effective hemostasis to control bleeding. In cases of inadvertent globe injury, the simulation emphasizes the importance of careful anatomical identification and proper instrument handling to minimize trauma. To prevent damage to surrounding tissues, the model includes scenarios that teach gentle dissection and the use of appropriate force during the procedure. By integrating these complications into the training, the simulation ensures that physicians are not only proficient in performing lateral canthotomy but also adept at recognizing and managing adverse events, thereby enhancing the overall safety and effectiveness of the procedure in real-life settings.

This newly presented lateral canthotomy simulation model provides a valuable tool in medical training. Furthermore, it was cost-effective to assemble and allowed the confidence of the trainees to be enhanced, so it may be one promising option for enhancing emergency procedural skills in resident doctors. The proposed enhancements addressing the current limitations could help further solidify its role in medical education and patient care outcomes.

## Conclusions

This study proposes a new model for the simulation of lateral canthotomy, which is low-cost and highly reusable. The construction cost is about 50 AED (13 USD), hence very suitable for resource-constrained medical institutions. It takes around 30 minutes to assemble this model, translating into hardly any cost in terms of labor and personnel. Participants detail an increase in levels of confidence and competence with the procedure after training with this model. While the current model lacks features such as bleeding in real-life situations, the affordability and effectiveness it presents compared to existing models exemplify its potential to democratize high-quality procedural training and improve patient outcomes during the most critical situations. As such, further developments should look into the incorporation of more realistic physiological responses and the expansion of the model's application to other surgical procedures. It is also further recommended that, based on its determined position in emergency medical training, longitudinal studies of the long-term effects of this tool on clinical performance and care provided for patients should be conducted.

## Figures and Tables

**Figure 9:**
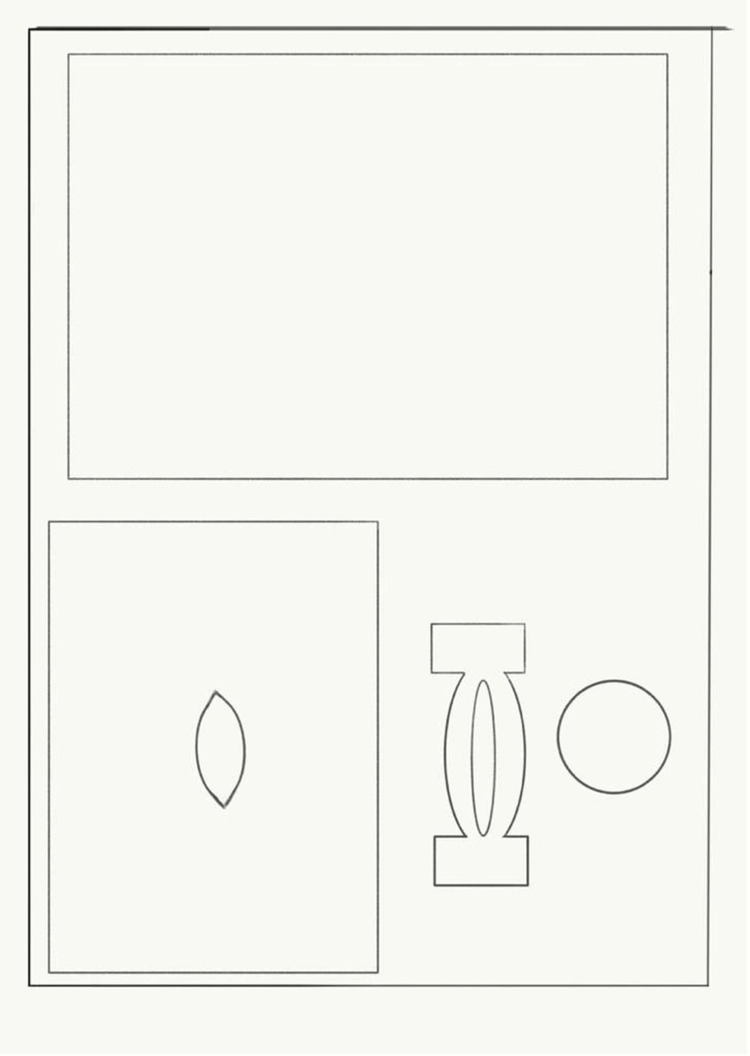
Lateral canthotomy simulation model template This template illustrates the shapes to be cut out for assembling the lateral canthotomy simulation model. The template should be prepared in A4 paper size. The cutout shapes include parts of the orbit, the lateral canthal ligament, skin layers, and the base. In this template, each of these shapes is given to a specific part of the model as described in the manuscript. It should be carefully cut out and assembled according to the instructions given in the technical report section of the study.

## Appendices

Appendix A 

**Table 3:** Survey questions on lateral canthotomy simulation model

Question number	Survey question
1	What is your age?
2	What is your gender?
3	What residency year are you in?
4	Have you practiced lateral canthotomy procedure before?
5	How comfortable are you performing the procedure? (1-5 scale)
6	How were the instructions to follow? (1-5 scale)
7	How long did the model assembly require? (in minutes)
8	How was the assembly of the model? (1-5 scale)
9	How would you rate re-using the model? (1-5 scale)
10	How comfortable are you with your skill to perform a lateral canthotomy procedure on a real patient? (1-5 scale)
11	Additional feedback (optional)

Appendix B
